# Pu‐erh tea extraction alleviates intestinal inflammation in mice with flora disorder by regulating gut microbiota

**DOI:** 10.1002/fsn3.2437

**Published:** 2021-07-19

**Authors:** Zhifang Zhang, Fei He, Weixing Yang, Li Yang, Siqi Huang, Hongling Mao, Yan Hou, Rong Xiao

**Affiliations:** ^1^ College of Food Science and Technology Yunnan Agriculture University Kunming China; ^2^ College of Biological Resource and Food Engineering Qujing Normal University Qujing China; ^3^ College of Longrun Pu‐erh Tea Yunnan Agriculture University Kunming China

**Keywords:** 16S rRNA, gut microbiota, intestinal inflammation, Pu‐erh tea extract

## Abstract

Pu‐erh tea is very popular in Southwestern China and South Asian countries and is now becoming increasingly popular in Europe due to its well‐documented beneficial effects on human health. Pu‐erh tea aqueous extracts can maintain intestinal homeostasis. However, the mechanism of its beneficial effects on intestinal flora disorder is not clear. In this study, we focused on the effects of ripe Pu‐erh tea aqueous extracts on the intestinal microbiota in an intestinal flora disorder mouse model. Physiological indexes and the tissue section staining results showed that feeding Pu‐erh tea extract could help mice regain weight and alleviate intestinal inflammation. Further assessment of the intestinal microflora found that Pu‐erh tea extract could promote the growth of intestinal probiotics and inhibit pathogenic bacteria, thereby achieving a treatment effect for enteritis. This study provides new evidence for the therapeutic effect of Pu‐erh tea.

## Graphical abstract

Pu‐erh tea is very popular in Southwestern China and South Asian countries, and now is becoming more and more popular in Europe because of its well‐documented beneficial effects on human health. This study explored the mechanism of Pu‐erh tea extract in promoting human health and improving intestinal flora disorder and found that Pu‐erh tea extract can alleviate the intestinal inflammation caused by antibiotics, reduce the degree of intestinal lesions, promote the growth of intestinal probiotics and inhibit pathogenic bacteria.

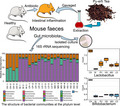

## 1 INTRODUCTION

Pu‐erh tea is a traditional Chinese tea that is particularly popular in Southwestern China and South Asian countries (Roda et al., 2019). Pu‐erh tea includes two varieties known as ripe Pu‐erh tea and raw Pu‐erh tea (Syu et al., 2008). Both varieties have been proved to decrease the levels of cholesterol and triglycerides (Hou et al., 2009), prevent lipid‐derived disorders (Cai et al., 2017), and alleviate obesity (Xia et al., 2019). These effects are associated with diverse mechanisms of action (Gao et al., 2018; Tu et al., 2016; Zeng et al., 2015). Ripe Pu‐erh tea is made from raw Pu‐erh tea through a unique “piling store” postfermentation technology and reduces production time. Considering the production costs, ripe Pu‐erh tea is the best choice for its antioxidant effects and antimicrobial properties (Roda et al., 2019).

The therapeutic effects of Pu‐erh tea are attributable to its bioactive components such as polyphenols, flavonoids, thearubigins, and caffeine content (Hou et al., 2009; Roda et al., 2019; Tu et al., 2016). After consumption, these components undergo microbial breakdown in the small intestine in the gastrointestinal tract and form new metabolites. Pu‐erh tea modulates the gut microbiome through its components and metabolites (Lee et al., 2006).

However, there are few studies on the prevention and treatment of enteritis and intestinal flora disorder by Pu‐erh tea at present, and the mechanism of its effect is not clear. To investigate the therapeutic effect of Pu‐erh tea on enteritis, we constructed an enteritis mouse model by intragastric administration of antibiotics. Pu‐erh tea extraction assisted weight recovery after feeding. Tissue sections further showed that Pu‐erh tea could help relieve intestinal inflammation in mice. This may be attributed to the increase in intestinal probiotics after Pu‐erh tea extract feeding in mice. To further confirm this speculation, we analyzed the mouse fecal bacterial community through high‐throughput 16S rRNA sequencing. It was found that Pu‐erh tea extract could promote the growth of probiotics in the intestinal tract of mice and inhibit pathogenic bacteria.

## 2 MATERIALS AND METHODS

### 2.1 Animals and antibiotic treatment

All murine experimental procedures were approved by the Animal Care and Use Committee of the Institute of Medical Biology, Chinese Academy of Medical Sciences, Kunming, China. All 78 8‐week‐old‐specific pathogen‐free (SPF) BALB/c female mice were randomly assigned to six groups with similar body weights in this study. The mice in the experimental group were intragastrically administered ampicillin (1.56 g/kg·bw) one time per day for 7 days and clindamycin (1.88 g/kg·bw) in drinking water to induce chronic enteritis. The mice in the control check (CK) group were treated with drinking water without antibiotics.

In the Ripe Pu‐erh tea intervention period, mice in the low‐dose (LD), medium‐dose (MD), and high‐dose (HD) groups were given extracts of 1.0, 2.0, and 3.0 g/kg·bw per day. The model (M) group and CK group were given drinking water instead. The mice in the positive control (PC) group were given live combined *Bacillus* *subtilis* and *Enterococcus* *faecium* enteric‐coated capsules (Beijing Hanmi Pharmaceutical Co., Ltd.). Body weight was determined every 3 days after the start of the Ripe Pu‐erh tea intervention period. After 21 days, venous blood samples and intestinal samples were taken after the mice were euthanized.

### 2.2 Aqueous extracts preparation

Ripe Pu‐erh tea was supplied by the Pu‐erh Tea Institute of China. The aqueous extract procedure was slightly modified based on a previous study (Roda et al., 2019). The tea samples were milled, and the powder was passed through a 20‐mesh sieve before use. Briefly, the tea powder was extracted with distilled water at 90°C for 40 min. The extracts were then filtered and concentrated at 60°C. Finally, the concentrates were sterilized and kept in bottles until use.

### 2.3 Determination of the Pu‐erh tea extracts content

Total polyphenols were analyzed by the modified Folin–Ciocalteu colorimetric method (Dewanto et al., 2002), according to the determination of total polyphenol and catechin contents in tea (GB/T 8313–2018). Briefly, 1 ml of extract or gallic acid calibration solutions (0–50 µg/ml) were added to a test tube with 5 ml of Folin–Ciocalteu reagent. The samples were mixed well and allowed to stand for 5 min. Then, 4 ml of 7.5% sodium carbonate aqueous solution was added. Samples were allowed to stand for 60 min at room temperature before measurement at 765 nm. The readings were compared to the gallic acid calibration curve. Based on Yemm et al. (1955), the free amino acid content was measured by the ninhydrin method, according to tea determination of free amino acid content (GB/T 8314–2013). The caffeine content was measured by automatic peak integration from LC‐UV analyses (Roda et al., 2019). Tea polysaccharides were analyzed by the anthrone sulfuric acid method, as described in a previous report (Teng et al., 2017).

### 2.4 Histological examination of the small intestine

Cross‐sections (4‐µm‐thick) of small bowel tissue were stained with hematoxylin and eosin after paraffin embedding. The samples were examined and photographed under a light microscope. Histopathological changes were evaluated in a double‐blind manner (blinded assessment of outcomes by two independent assessors). Each section of ischemic intestinal tissue was scored on a 6‐point scale as described by (Chiu et al., 1970).

### 2.5 Determination of cytokine mRNA by qRT‐PCR

The small intestine samples were frozen in liquid nitrogen and stored at –80°C. Total RNA was extracted as previously described (Liu et al., 2016) and converted to cDNA by a reverse transcription kit (Thermo Fisher Scientific Inc). For the determination of cytokine mRNA, we performed qRT‐PCR using specific primers and SYBR Green qPCR Master Mix. *GAPDH* was used as the internal control.

### 2.6 White blood cell count

Blood samples were collected from the caudal vein of each group. A Mindray BC‐2800vet hematology analyzer (Mindray) was used to determine the white blood cell (WBC) count.

### 2.7 DNA extraction and microbiota analysis

DNA was isolated by phenol–chloroform extraction as previously described (Wang et al., 2016). Briefly, approximately 20 mg of each sample was mixed with 0.2 g zirconia beads, 700 μl lysis buffer, and 250 μl phenol:chloroform:isoamyl alcohol (25:24:1; v/v) in a 2 ml screw‐cap tube and homogenized (Turnbaugh et al., 2009). Then 250 μl of 10 M ammonium acetate was added to the mixture, and the mixture was incubated for 5 min on ice and then centrifuged. The supernatant was treated twice with 250 μl phenol:chloroform:isoamyl alcohol (25:24:1; v/v) and twice with 250 μl chloroform. Next, the DNA extract was precipitated with centrifugation by adding prechilled isopropanol in a 1:1 ratio, washed twice with 1 ml 70% (v/v) prechilled ethanol and dried. Finally, the extract was dissolved in deionized water, and DNA‐free RNase (10 mg/μl) was added to remove any remaining RNA.

The 16S rDNA V3‐V4 region was amplified using a KAPA LTP library preparation kit (Kapa Biosystems, Inc) according to the manufacturer's protocol, with primers and adapters for HiSeq 2,500 PE250 sequencing. The forward primer was 341F (5′‐ACTCCTACGGGRSGCAGCAG‐3′), and the reverse primer was 806R (5′‐GGACTACHVGGGTWTCTAAT‐3′) (Hugerth et al., 2014). The library quality was assessed using an Agilent Bioanalyzer 2,100 system and Qubit 2.0 Fluorometer (Life Technologies). The prepared libraries were sequenced with the Illumina Hiseq 2,500 platform to obtain 250 bp paired‐end reads. The data were assembled with PANDAseq software (Masella et al., 2012) and checked to ensure that the sequence matched completely with the index sequences and had no more than one mismatch error present in the primer sequences. The filtered data were analyzed using the QIIME pipeline as previously reported (Caporaso et al., 2010). The characterization of microorganismal features differentiating the fecal microbiota was performed using the linear discriminant analysis effect size (LEfSe) method for biomarker discovery, which emphasizes both statistical significance and biological relevance (Segata et al., 2011). The sequencing data were deposited in the NCBI Sequence Read Archive database, and the accession number was SRP228277. Biodiversity indexes such as the Shannon index, coverage ratios, and Veen diagrams were calculated using Mothur (Schloss et al., 2009) and ggplot2 (Wickham, 2009).

### 2.8 Bacterial culture of feces and identification of predominant bacteria

Standard plate counts were performed for the cultivation of aerobic, enteric, coliform, and lactic acid bacteria from fresh fecal samples. Briefly, 0.5 g intestinal content samples from each group were serially diluted and seeded onto TPY agar (for *Bifidobacterium*), Pfizer agar (for *Enterococcus*), VRBGA agar (for *enterobacterium*), and MRS agar (for *Lactobacillus*). All plates were incubated for 24–48 hr at 37°C. All samples were assayed in duplicate.

### 2.9 Statistical analysis

Data were analyzed with analysis of variance (ANOVA) with the *post hoc* Tukey test for multiple comparisons. A *p* value of .05 was the threshold for significance.

## 3 RESULT

### 3.1 Main aqueous extracts of Pu‐erh tea

Pu‐erh tea was extracted according to the aqueous extract procedure for feeding during the ripe Pu‐erh tea intervention period. The contents of total polyphenols, free amino acids, caffeine, and tea polysaccharides are listed in Table 1.

**TABLE 1:** Content of chemical components in Pu‐erh tea extract

H_2_O (%)	Extract (%)	Polysaccharide (%)	Polyphenol (%)	Caffeine (%)	Free amino acids (%)
9.60 ± 0.56	28.70 ± 0.28	2.14 ± 0.08	9.80 ± 0.42	9.25 ± 0.02	2.55 ± 0.07

### 3.2 Pu‐erh tea extract helps the recovery of body weights and serum blood profiles in the chronic enteritis mouse model

Before antibiotic feeding, the mouse hair was smooth and shiny, and the feces were dry and light yellow. After 7 days of antibiotic gavage, the stools of the mice became mucous or watery, and the hair became irregular, which indicates an imbalance in the intestinal flora.

After antibiotic feeding, all of the mice lost weight due to the intragastric administration. However, the weight of the CK group was significantly higher than that of the other groups (*p* < .05; Table 2). During the Ripe Pu‐erh tea intervention period, the mice in the HD and PC groups had a faster weight recovery than the other groups, which may be due to the curative effects of live probiotics or the ripe Pu‐erh tea concentrates.

**TABLE 2:** The changes in body weight of each group

Group	Weight change after modeling (g)	Change ratio after modeling (%)	Weight change after intervention	Change ratio after intervention (%)
CK	−0.28 ± 0.13^b^	−1.42 ± 0.64^b^	0.22 ± 0.19^d^	1.13 ± 1.00^b^
M	−0.61 ± 0.53^ab^	−3.18 ± 2.84^ab^	0.56 ± 0.34^bc^	2.86 ± 1.65^ab^
CP	−0.76 ± 0.38^a^	−3.38 ± 2.14^ab^	0.93 ± 0.47^a^	4.35 ± 2.32^a^
LD	−0.65 ± 0.37^ab^	−3.31 ± 1.83^ab^	0.34 ± 0.24^cd^	1.74 ± 1.22^b^
MD	−0.81 ± 0.40^a^	−4.37 ± 2.25^a^	0.39 ± 0.28^cd^	2.08 ± 1.53^ab^
HD	−0.83 ± 0.38^a^	−4.37 ± 2.00^a^	0.73 ± 0.39^ab^	3.63 ± 1.79^a^

Note

Change ratio after modeling (%) = weight change after modeling/weight after modeling *100; change ratio after intervention (%) = (weight after modeling ‐weight after intervention)/weight after intervention *100.

Different letters in the same column indicate significant differences (*p* < .05), conversely indicates not significant (*p* < .05).

### 3.3 Intestinal inflammation of the small intestine is alleviated by Pu‐erh tea extract

**FIGURE 1:**
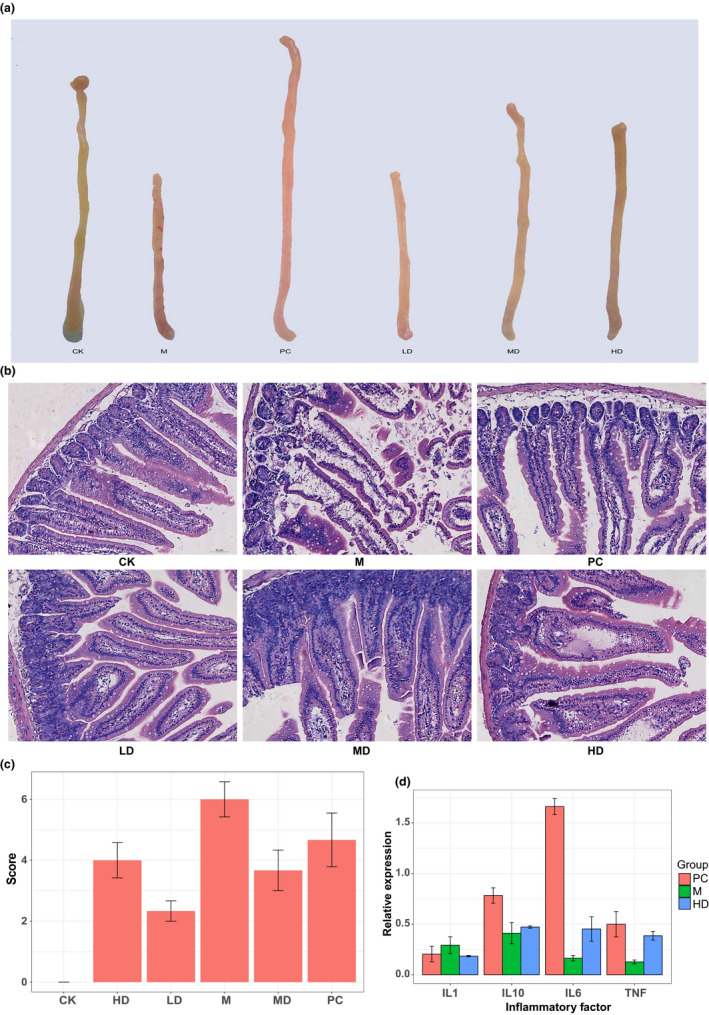
Pu‐erh tea extract alleviates inflammation in the small intestine. (a) Observation of the small intestine from each group. Pu‐erh tea extraction improved the shape of the small intestine. (b) Pathological slices of the small intestine from each group. (c) The scores of pathological slices. The scores were evaluated in a double‐blind manner by two independent assessors. (d) Determination of cytokine mRNA expression by qRT‐PCR

After the ripe Pu‐erh tea intervention period, the mice were sacrificed, and the small intestines were removed for histological scoring. The small intestine of mice in the CK group was light yellow with a thick wall. In the M group, the wall of the small intestine was congested, swollen, thinned, and thickened with lymph nodes (Figure 1a). The appearance of small intestine tissues in the MD and HD groups was the closest to that of the CK group (Figure 1a).

The small intestine of all groups of mice had different degrees of pathological changes except the CK group. Mice in the M group showed more severe histological damage (severe edema of mucosal villi accompanied by severe intestinal crypt injury and a large number of disintegrated intestinal villi) than the other groups (Figure 1b).

The histological scores were evaluated in a double‐blind manner (Figure 1c). The small intestine of all groups of mice had different degrees of pathological changes except the CK group. Compared with the M group, the Chiu's score of the LD, MD, and HD groups decreased significantly (*p* < .05), indicating that Ripe Pu‐erh tea has a protective effect against intestinal tissue damage in the diarrhea model mice. Compared with the CK group, the Chiu's score of the LD, MD, HD groups increased significantly (*p* < .05), as the mice did not recover sufficiently for 2 weeks. Compared with the PC group, the Chiu's score of the LD, MD, HD groups showed no significant difference (*p* > .05), which indicates that the Ripe Pu‐erh tea concentrates has curative effects similar to live probiotics.

Since different degrees of pathological changes were observed in the small intestine of each experimental group through histological examination (H&E) staining, to accurately determine the levels of inflammation, qRT‐PCR technology was used to accurately determine and analyze cytokine expression at the mRNA level. The results showed that antibiotic feeding also induced immune cells in the mice compared with the CK group (*p* < .05), indicating that the model of enteritis and intestinal disorder was successfully established by using antibiotics and that the inflammatory level in the antibiotic group was significantly increased (Figure 1d). Similar to the weight data, the number of immune cells in the HD and PC groups recovered faster (*p* < .05; Table 3).

**TABLE 3:** The immune cell count of the mice in each group

Group	Monocyte 10^9^/L			Granulocyte 10^9^/L		
	Initial	After modeling	After intervention	Initial	After modeling	After intervention
CK	0.30 ± 0.07^a^	0.31 ± 0.09^a^	0.37 ± 0.13^a^	2.67 ± 0.97^a^	3.17 ± 0.55^a^	3.93 ± 0.70^a^
M	0.37 ± 0.06^a^	0.20 ± 0.00^ab^	0.23 ± 0.06^a^	3.10 ± 0.26^a^	3.15 ± 0.21^a^	2.63 ± 0.71^ab^
CP	0.27 ± 0.12^a^	0.20 ± 0.00^ab^	0.23 ± 0.06^a^	2.70 ± 0.44^a^	2.83 ± 0.51^a^	2.80 ± 0.44^ab^
LD	0.30 ± 0.00^a^	0.10 ± 0.10^b^	0.20 ± 0.00^a^	3.03 ± 0.15^a^	2.23 ± 0.25^a^	2.43 ± 0.93^bc^
MD	0.23 ± 0.15^a^	0.17 ± 0.06^ab^	0.23 ± 0.06^a^	2.53 ± 0.71^a^	2.70 ± 0.78^a^	2.33 ± 0.15^c^
HD	0.27 ± 0.06^a^	0.17 ± 0.06^ab^	0.23 ± 0.06^a^	3.40 ± 0.10^a^	2.80 ± 0.62^a^	3.13 ± 1.31^ab^

Note

Values are mean ± S.E.M. Data in the same line with different letters are significantly different (*α* < 0.05) as identified by a post hoc Duncan's test.

Different letters in the same column indicate significant differences (*p* < .05), conversely indicates not significant (*p* < .05).

### 3.4 Diversity of fecal microbiota in mice as revealed by 16S rRNA high‐throughput sequencing

**FIGURE 2:**
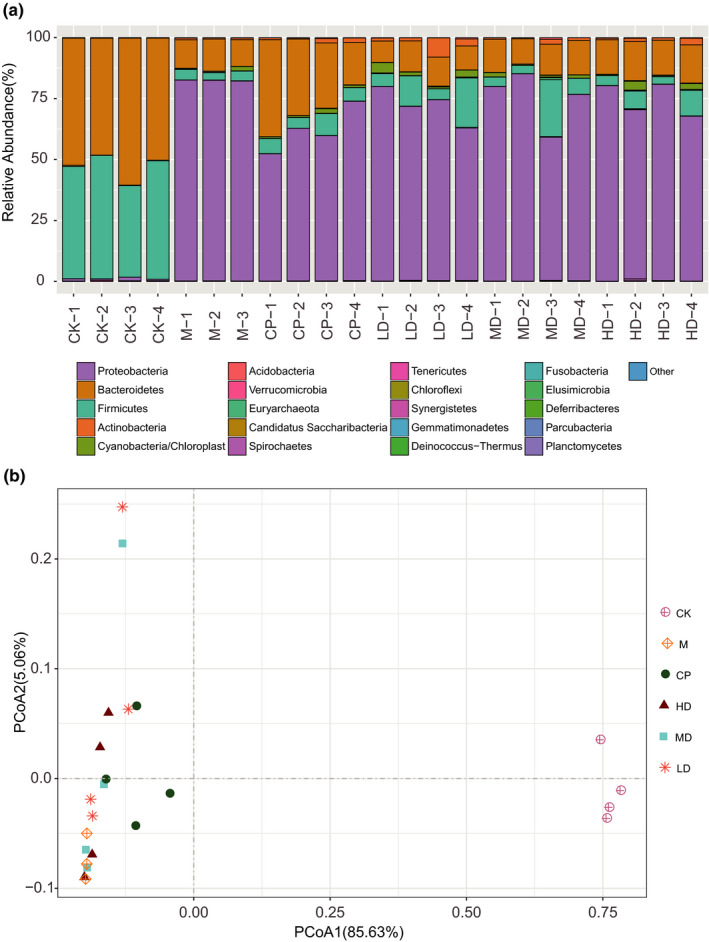
Bacterial communities in mouse intestinal flora from each group by Illumina sequencing of 16S rRNA amplicons. (a) The structure of bacterial communities at the phylum level. (b) Grouping of mouse intestinal flora based on principal coordinates analysis

To further analyze the diversity of microflora among the groups, 16S rRNA high‐throughput sequencing was used for detection. The groups had high diversity at the phylum classification level, and the most abundant phyla were *Firmicutes*, *Proteobacteria,* and *Bacteroidetes*. LD, MD, HD, and PC groups significantly increased the abundance of *Firmicutes* compared with the M group (*p* < .05), while the abundance of the LD group was higher than that of the HD group (*p* < .05). In addition, the LD, MD, HD, and PC groups had significantly decreased abundances of *Proteobacteria* compared with the M group (*p* < .05). Antibiotics significantly reduced the proportion of intestinal *Bacteroidetes* in mice (Figure 2a).

To characterize the global differences in fecal microbial communities between groups, a principal coordinates analysis (PCoA) was performed on unweighted UniFrac distances. A significant separation was shown between the CK group and the other groups. The results also showed that the feeding of live probiotics significantly changed the structure of the rat fecal bacterial communities. Pu‐erh tea extract influenced the structure of the rat fecal bacterial communities, but the dominant microbiota did not change (Figure 2b).

Using a 97% sequence similarity cut‐off, the total operational taxonomic units (OTU) number detected in the fecal microbial communities of the M group was 389.33 ± 16.5. The OTU number in the mice fed a low, medium or high dose of extract (436.25 (±145.73), 540.00 (±248.35) and 484.75 (±158.81), respectively) were higher than those detected in the CK groups (226.25 ± 7.89). The Pu‐erh tea extraction feeding groups had more unique OTUs than the others, as the unique OTUs of the LD, MD, and HD groups were 214, 239, and 200, respectively.

The Shannon index values ranged from 0.66 to 0.94. The abundance of the CK group was significantly higher than that of the other groups. Interestingly, the low dose of the extract improved the fecal microbial communities, while the high dose decreased the abundance (Table 4). This may be because the extract has a bacteriostatic effect.

**TABLE 4:** The intestinal microbial diversity of the mice in each group

Group	Alpha diversity				
	Chao1	Observed‐species	Shannon	Simpson	Coverage
CK	257.95 ± 13.22^bc^	229.00 ± 5.77^bc^	5.24 ± 0.42^a^	0.94 ± 0.03^a^	1.00
M	443.50 ± 45.79^ab^	385.33 ± 14.19^ab^	3.34 ± 0.11^b^	0.66 ± 0.00^d^	1.00
CP	425.62 ± 61.59^ab^	377.50 ± 76.38^ab^	4.20 ± 0.38^b^	0.80 ± 0.04^bc^	1.00
LD	454.58 ± 157.59^ab^	438.50 ± 153.29^a^	4.22 ± 0.82^b^	0.73 ± 0.08^cd^	1.00
MD	569.08 ± 258.17^a^	494.25 ± 231.96^a^	3.96 ± 1.13^b^	0.71 ± 0.11^cd^	1.00
HD	536.39 ± 154.41^a^	481.75 ± 158.29^a^	3.89 ± 0.67^b^	0.71 ± 0.06^cd^	1.00

Note

Different letters in the same column indicate significant differences (*p* < .05), conversely indicates not significant (*p* < .05).

### 3.5 LefSe analysis of bacterial community structures in the feces of mice

**FIGURE 3:**
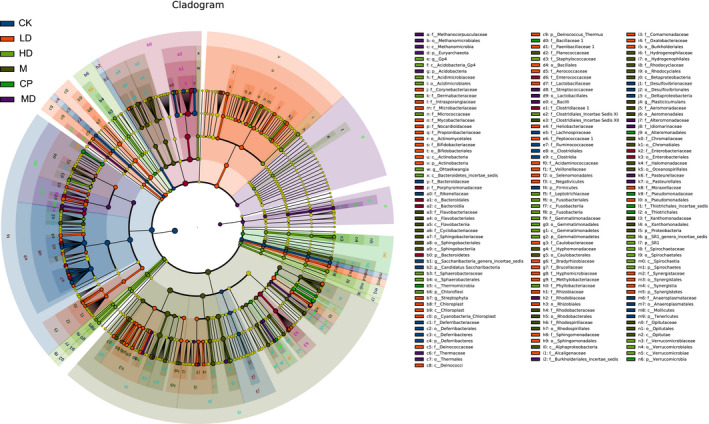
Linear discriminant analysis (LDA) coupled with effect size measurements identifies Proteobacteria as the most differential phylum in mouse gut microbiology by 16S rRNA sequencing

LEfSe was used to detect class discriminating OTUs or bacterial phylotypes (from phylum to family level) in the different feeding groups. The *Proteobacteria* abundance of the M group was significantly higher than that of the other groups, which induced severe diarrhea and sepsis in the animals (Figure 3) (Kaakoush et al., 2012). The *Lactobacillales*, *Lactobacillaceae*, and *Lactobacillus* abundances of the Pu‐erh tea extract feeding group were significantly higher than those of the other groups, which have broad‐spectrum antimicrobial activity (Mohanty et al., 2019; Spinler et al., 2017).

### 3.6 Pu‐erh tea extract contributes to the recovery of probiotics in the small intestine

**FIGURE 4:**
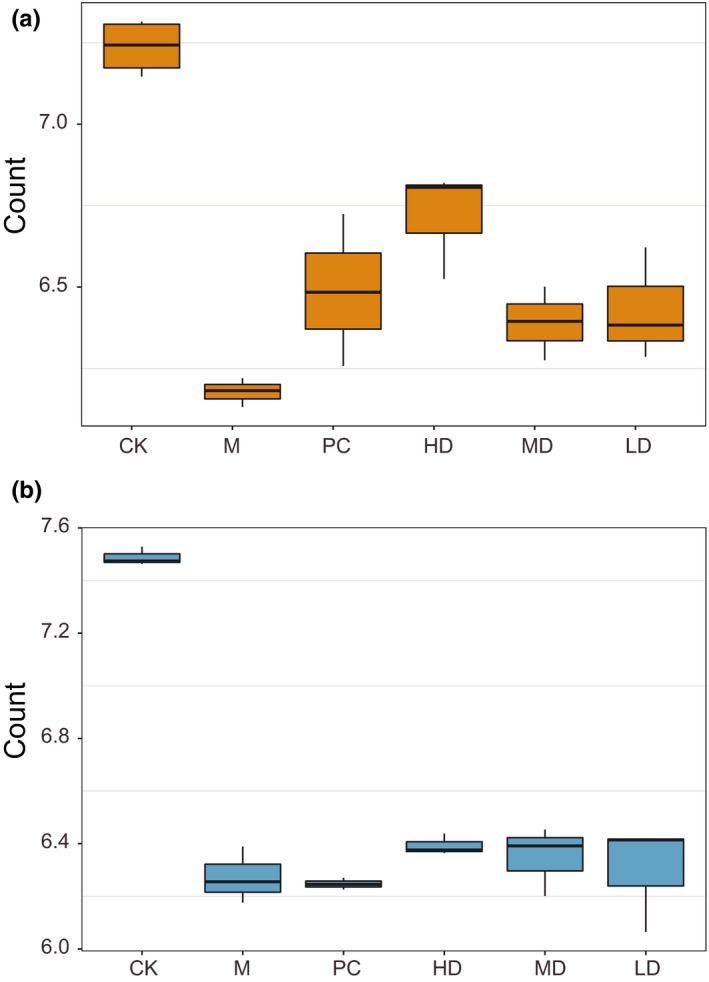
Pu‐erh tea extract helps recovery of probiotics in the small intestine. (a) The relative abundance of Lactobacillus. (b) The relative abundance of Bifidobacterium

Considering that the relative abundance of *Lactobacillus* and other probiotics in the intestinal flora of mice fed Pu‐erh tea was significantly increased, as determined by 16S rRNA technology, to accurately determine the colonization quantity of probiotics in the intestine, the predominant probiotics including *Lactobacillus* and *Bifidobacterium*, in the intestinal contents of each group were counted. CK group had the most abundant probiotics (Figure 4). The mice fed live probiotics or ripe Pu‐erh tea concentrates had more *Lactobacillus* and *Bifidobacterium* than the M group (*p* < .05) but significantly less than the CK group (*p* < .01; Figure 4a,b). These results indicated that Pu‐erh tea can significantly promote the proliferation of *Bifidobacterium* and *Lactobacillus* in the intestinal tract, and alleviate intestinal disorders and injury by increasing the number of probiotics in the intestine.

## 4 DISCUSSION

Pu‐erh tea is very popular worldwide due to its unique flavor and health effects (Xia et al., 2019). The tea extracts have been proved to influence the microbial structure. An in vitro experiment confirmed that the tea extracts have an antimicrobial effect, and their impact on the microbial community was also verified (van Dorsten et al., 2012). Several studies in animals stated that tea extracts increase probiotics in the gut, such as *Lactobacillus* spp. and *Akkermansia muciniphila* (Hara et al., 1995; Xia et al., 2019), which may help maintain intestinal homeostasis (Sun & Jia, 2018). The tea extracts contain a complex blend of chemical compounds, such as polyphenols, free amino acids, caffeine, and polysaccharides. These components may help to improve the structure of the intestinal flora. Many studies have shown that polyphenols can decrease proinflammatory microbes and counteract disease‐induced dysbiosis in colitis models (Zhao & Jiang, 2021). Moreover, the bioactive phenol is considered to be the main component of Pu‐erh tea responsible for its health‐promoting function (Etxeberria et al., 2013). Caffeine can also inhibit the growth of nonprobiotics such as *Escherichia coli* (Sales et al., 2020), and polysaccharides are thought to stimulate probiotic bacterial growth.

In this study, the effects of aqueous extracts of ripe Pu‐erh tea on the intestinal microbiota in the intestinal flora were evaluated in a flora disorder mouse model. Antibiotics upset the balance of intestinal flora in mice, leading to intestinal inflammation, which affects appetite and food absorption. Although the mice in each group had a similar dietary intake during the ripe Pu‐erh tea intervention period, the PC group and the groups that were given the tea extract had a faster rate of weight recovery. Both live probiotics and the tea extract could correct the dysbiome by increasing the diversity of intestinal flora, which may alleviate deviant inflammation. The histological score also showed that Pu‐erh tea extract alleviated intestinal inflammation, which supports this speculation.

To further evaluate the effect of Pu‐erh tea on the intestinal microorganisms of mice, we first counted the *Lactobacillus* and *Bifidobacterium* in each group. These two probiotics have been proved to prevent antibiotic‐associated diarrhea via restoration of the gut microflora (Goldenberg et al., 2015, Hayes & Vargas, 2016). As expected, the mice fed with tea extract had higher counts than the M group. We also evaluated the diversity of the fecal microbiota by 16S rRNA high‐throughput sequencing. The *Proteobacteria* level in the M group was significantly higher than that of the other groups, which was considered related to the lipopolysaccharides (Cai et al., 2018; Chassaing & Gewirtz, 2018; Zhu et al., 2019). The lipopolysaccharides that the *Proteobacteria* spp. produce induce severe diarrhea and intestinal inflammation (Asami et al., 2017; Qi et al., 2017; Yuan et al., 2019). Compared with the M group, the Pu‐erh tea extract feeding groups had a higher abundance of *Lactobacillales*, *Lactobacillaceae*, and *Lactobacillus*. These strains have broad‐spectrum antimicrobial activity (Mohanty et al., 2019; Spinler et al., 2017). Much of the research has shown that these bacteria can inhibit proinflammatory gene expression, regulate intestinal epithelial barrier function, and restore intestinal microorganisms (Asgari et al., 2018; Morita et al., 2018; Sun et al., 2018; Zhou et al., 2018). Other works focusing on the treatment of intestinal inflammation have also shown similar changes in the intestinal flora (Yuan et al., 2019; Zhu et al., 2019).

In summary, we demonstrated that Pu‐erh tea extraction could help weight recovery and alleviate intestinal inflammation in antibiotic‐associated intestinal inflammation by increasing the microbial community biodiversity of the intestine.

## CONFLICT OF INTEREST

The authors declare that they do not have any conflict of interest.

## AUTHOR CONTRIBUTIONS

**Zhifang Zhang:** Conceptualization (lead); Writing‐original draft (equal); Writing‐review & editing (equal). **Fei He:** Conceptualization (equal); Data curation (equal); Writing‐original draft (equal); Writing‐review & editing (equal). **Weixing Yang:** Resources (equal). **Li Yang:** Visualization (equal). **Siqi Huang:** Visualization (equal). **Hongling Mao:** Resources (equal). **Yan Hou:** Funding acquisition (equal); Methodology (equal). **Rong Xiao:** Funding acquisition (equal); Methodology (equal).

## ETHICAL APPROVAL

All murine experimental procedures were approved by the Animal Care and Use Committee of the Institute of Medical Biology, Chinese Academy of Medical Sciences, Kunming, China.

## Data Availability

All data used during the study are available from the corresponding author on request. The sequencing data were deposited in the NCBI Sequence Read Archive database, and the accession number was SRP228277.
